# Specific Binding and Characteristics of 18β-Glycyrrhetinic Acid in Rat Brain

**DOI:** 10.1371/journal.pone.0095760

**Published:** 2014-04-21

**Authors:** Kazushige Mizoguchi, Hitomi Kanno, Yasushi Ikarashi, Yoshio Kase

**Affiliations:** Tsumura Research Laboratories, Kampo Scientific Strategies Division, Tsumura & Co., Yoshiwara, Ami-machi, Inashiki-gun, Ibaraki, Japan; Glasgow University, United Kingdom

## Abstract

18β-Glycyrrhetinic acid (GA) is the aglycone of glycyrrhizin that is a component of Glycyrrhiza, and has several pharmacological actions in the central nervous system. Recently, GA has been demonstrated to reach the brain by crossing the blood-brain barrier in rats after oral administration of a Glycyrrhiza-containing traditional Japanese medicine, yokukansan. These findings suggest that there are specific binding sites for GA in the brain. Here we show evidence that [^3^H]GA binds specifically to several brain areas by quantitative autoradiography; the density was higher in the hippocampus, moderate in the caudate putamen, nucleus accumbens, amygdala, olfactory bulb, cerebral cortex, thalamus, and mid brain, and lower in the brain stem and cerebellum. Several kinds of steroids, gap junction-blocking reagents, glutamate transporter-recognized compounds, and glutamate receptor agonists did not inhibit the [^3^H]GA binding. Microautoradiography showed that the [^3^H]GA signals in the hippocampus were distributed in small non-neuronal cells similar to astrocytes. Immunohistochemical analysis revealed that immunoreactivity of 11β-hydroxysteroid dehydrogenase type-1 (11β-HSD1), a defined molecule recognized by GA, was detected mainly in neurons, moderately in astrocytes, and very slightly in microglial cells, of the hippocampus. These results demonstrate that specific binding sites for GA exist in rat brain tissue, and suggest that the pharmacological actions of GA may be related to 11β-HSD1 in astrocytes. This finding provides important information to understand the pharmacology of GA in the brain.

## Introduction

**Figure 1:**
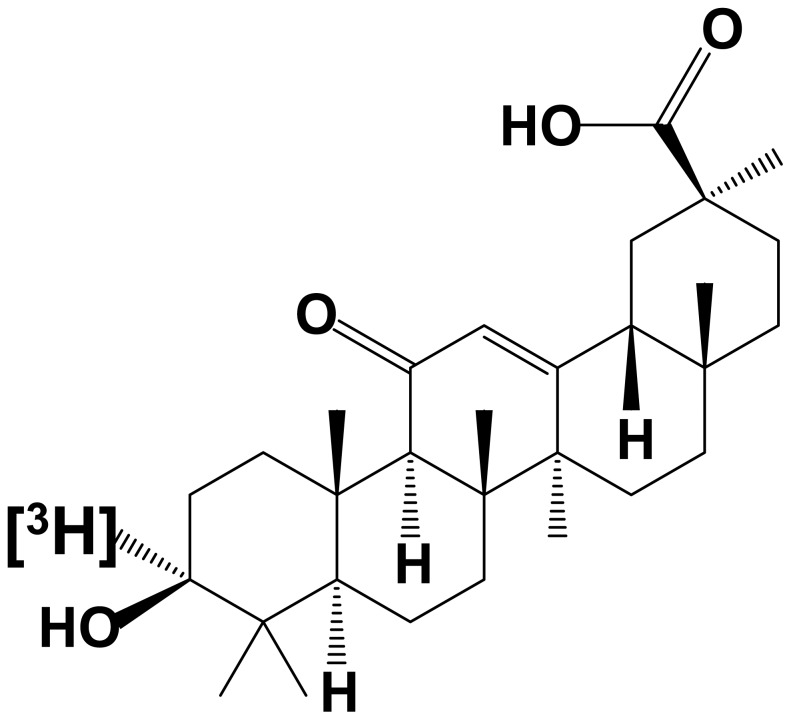
Chemical structure of tritium-labeled 18β-glycyrrhetinic acid ([3-^3^H]GA), which is a triterpenoid having a ketone group at the 11^th^ position.[^3^H] was introduced at the 3^rd^ position in its structure.

18β-Glycyrrhetinic acid (GA) is a triterpenoid having a ketone group at the 11^th^ position in its structure (chemical structure shown in Figure 1). GA is the aglycone of glycyrrhizin that is a component of Glycyrrhiza, also called licorice root, and one of the most common drugs used clinically. It is well known that orally administered glycyrrhizin is metabolized to GA by β-glucuronidase activity of intestinal flora [1], and thereafter, GA is absorbed from the small intestine into the systemic circulation. GA as well as glycyrrhizin has various pharmacological actions such as anti-inflammatory, anti-allergic, anti-gastric ulcer, anti-hepatitis, and anti-hepatotoxic activities [2]. Although the effects of GA in the central nervous system (CNS) are not fully understood, several reports have shown that GA derivatives have ameliorative effects in a rodent model of ischemia/reperfusion brain injury [3], experimental autoimmune encephalomyelitis [4], and amyotrophic lateral sclerosis and Alzheimer's disease [5]. These findings strongly suggest that GA can interact with some molecules mediating its pharmacological actions in the CNS. Indeed, GA has been reported to bind to mineralocorticoid receptors (MRs) [6], 11β-hydroxysteroid dehydrogenase-1 (11β-HSD1) [7], and gap junctions [8], and these molecules exist in several types of cells in the brain. However, there is no evidence that GA interacts with these molecules in the brain. On the contrary, it was unclear whether GA can bind specifically to brain tissue.

Glycyrrhiza has also long been used as a raw material in mixed herbal medicines. One such medicine is yokukansan, which is a traditional Japanese (Kampo) medicine, and consists of seven medicinal herbs including Glycyrrhiza. This medicine has been approved by the Ministry of Health, Labour, and Welfare of Japan as a remedy for neurosis, insomnia, and night crying and irritability in children. Recently, clinical trials have demonstrated that yokukansan improves behavioral and psychological symptoms of dementia (BPSD), i.e., aggressiveness, agitation, anxiety, hallucination, sleep disturbance, and psychotic disorders, observed in several types of dementia including Alzheimer's disease without serious adverse effects [9], [10], [11], [12]. Several lines of evidence have suggested that attenuation of glutamate-induced neural excitation and neurotoxicity is the mechanism underlying these effects. For example, yokukansan reduces glutamate neurotransmission in the hippocampus of zinc- or thiamine-deficient rats, animal models of aggressiveness, excitability, and anxiety [13]–[14]. Among a large number of components included in this medicine, GA has been identified as a candidate compound involved in yokukansan's actions. For example, GA facilitates glutamate uptake into astrocytes through increased glutamate transport activity in cultures [15]. GA also ameliorates glutamate-induced neurotoxicity in primary cultured cortical neurons [16]. On the other hand, recent basic studies have suggested that GA is absorbed into the blood after oral administration of yokukansan, and then reaches the brain by crossing the blood-brain barrier [17]. Generally, compounds having pharmacological actions express those actions by interacting with target molecules such as receptor, channel, enzyme, and so on. However, the target molecule(s) of GA in the brain remains unclear, and it is even unknown whether GA can specifically bind to brain tissues. Thus, it is very important to clarify the binding characteristics of GA in the brain to explore the pharmacological mechanisms of yokukansan.

Therefore, identification and characterization of GA binding sites in the brain would facilitate understanding of the neuropharmacological actions of GA. The present study was designed to identify the GA binding sites in rat brain by quantitative autoradiographic and microautoradiographic analyses using tritium-labeled GA ([^3^H]GA).

## Materials and Methods

### Synthesis of [^3^H]GA

[3-^3^H]GA was synthesized from unlabelled GA purchased from Sigma-Aldrich (St. Louis, MO, USA) by Quotient Bioresearch (Radiochemicals) Ltd (Cardiff, UK). In brief, GA was oxidized to produce 3-keto GA according to the method of [18], and the resultant product was reduced with tritium-labeled sodium borohydride to generate [3-^3^H]GA (see Figure 1). The relative radioactivity of [^3^H]GA was 444 GBq/mmol, and the purity was 99.8%.

### Animals

Naive seven-week-old male Wistar rats were purchased from Charles River Laboratories Japan (Kanagawa, Japan). They were housed three or five per cage in a temperature (23±3°C)-, relative humidity (40–70%)- and light (12 h light/dark schedule; lights on at 7:00 a.m.)-controlled environment and were fed laboratory food and water *ad libitum*.

### Ethics statement

This study was carried out in accordance with the recommendations in the Guide for the Care and Use of Laboratory Animals of the Japanese Association for Laboratory Animal Science. The protocol was approved by the Committee on the Ethics of Animal Experiments of Nemoto Science & Co (Number: 13–275) and Tsumura & Co. All surgery was performed under sodium pentobarbital anesthesia, and the experiments in the present study were designed to minimize the number of animals used.

### Quantitative autoradiography

The animals were killed by decapitation, and the brain was quickly removed, immediately frozen in powdered dry ice, and stored at −80°C. Then, sections (15 µm) were cut using a freezing microtome in the coronal or sagittal plane, mounted on gelatin-coated slide glass, and stored at −80°C. The following coordinates relative to bregma were used for the coronal plane: anteroposterior +4.00 and −3.30 and the sagittal plane: lateral +1.90 and +0.18. On the day of the experiment, sections were thawed, rinsed with 50 mM potassium phosphate buffer, pH 7.4, containing 0.01% ascorbic acid, and incubated with [^3^H]GA in the same buffer at 4°C for 16 h, followed by 2×30 min washes in ice-cold buffer. The sections were then dipped for a few seconds in ice-cold distilled water to remove salts and dried under air. For saturation-binding assay, the employed concentrations of [^3^H]GA ranged from 0.25 nM to 75 nM. The non-specific binding of [^3^H]GA was determined by incubation of parallel sections in the presence of 50 µM unlabeled GA.

Radiolabeled, dried tissue sections were apposed to a tritium-sensitive imaging plate (BAS IP TR 2040 E; Fujifilm Corp., Tokyo, Japan) along with [^3^H]Micro-scales (Higher Activity Range, 51.3 to 1252.0 Bq/mg; GE Healthcare UK Ltd., Bucks, UK) for 16 h. After exposure, the plates were automatically analyzed in an imaging analyzer (Typhoon FLA 7000; GE Healthcare) to generate autoradiograms, and the radioactivity of [^3^H]GA in each brain region was measured. The identification and nomenclature of brain structures were based on the rat brain atlas of Paxinos and Watson [19]. In addition, reference sections were stained with cresyl violet to confirm the localization of [^3^H]GA binding sites on the autoradiograms. The number of binding sites was calculated from the radioactivity values referring the calibration curve generated from a [^3^H] standard co-exposed with the tissue sections. In several brain regions, densitometric readings were collected bilaterally from series of sections and averaged. Specific binding (SB) was calculated by subtracting the values of non-specific binding (NSB) from those of total binding (TB). The saturation binding data were analyzed by a Scatchard plot, and the maximal number of binding site (*B*_max_) and equilibrium dissociation constant (*K*_d_) were calculated. The results were expressed as pmol [^3^H]GA bound/mg wet tissue for *B*_max_ and nM for *K*_d_.

For the competition-binding assay, the sections were incubated with 20 nM [^3^H]GA at 4°C for 16 h in the presence of each reagent at 20 µM as follows: corticosterone (agonist for glucocorticoid receptor (GR) and MR), aldosterone (MR agonist), dexamethasone (GR agonist), allopregnanolone (neurosteroid receptor agonist), pregnenolone (neurosteroid receptor agonist), dehydroepiandrosterone (neurosteroid receptor agonist), lanthanum chloride heptahydrate (La^3+^; blocker of gap junction connexin hemichannels including Cx43) [20], probenecid (inhibitor of gap junction pannexin 1 hemichannels) [21], dihydrokainic acid (DKA, glutamate transporter 1 (GLT-1) inhibitor), DL-threo-*β*-hydroxyaspartic acid (TBHA, non-specific glutamate transporter inhibitor), and glutamate receptor agonists, i.e., N-methyl-D-aspartic acid (NMDA), kainic acid (KA), and α-amino-3-hydroxy-5-methyl-4-isoxazolepropionic acid (AMPA).

### Emulsion microautoradiography

Emulsion microautoradiography was performed using tissue sections reacted with 50 nM [^3^H]GA in quantitative autoradiography described above. Thus, the sections were dipped in photo emulsion (NTB 2; Eastman-Kodak, Rochester, NY, USA), and exposed for four weeks. The sections were then developed in Kodak D-19 developer (Eastman-Kodak), fixed in Kodak rapid fixer, and counter stained with hematoxylin. The silver grains in the corresponding brain regions were observed under a microscope connected to a digital camera (DM2000 and DFC295; Leica Microsystems, Wetzlar, Germany), and the data were analyzed using the accompanying software (Leica Application Suite 3.6.0; Leica Microsystems).

### Immunohistochemistry

Glial fibrillary acidic protein (GFAP), ionized calcium-binding adaptor molecule 1 (Iba1), and 11β-HSD1 were immunostained using standard immunohistochemical techniques. Briefly, the rats were perfused transcardially with 0.1 M phosphate buffer (pH 7.4), followed by 4% paraformaldehyde in 0.1 M phosphate buffer under pentobarbital anesthesia (50 mg/kg, i.p.). The brains were post-fixed overnight at 4°C in the same fixative.

For bright field immunohistochemistry for GFAP, the brains were dehydrated and embedded in paraffin. Coronal sections (5 µm) were deparaffinized and rehydrated. Endogenous peroxidase activity was blocked with 0.3% hydrogen peroxide in methanol, followed by incubation with 2× Block Ace solution (Dainippon Pharmaceutical, Osaka, Japan) and with 10% normal donkey serum (NDS). The sections were incubated overnight with rabbit polyclonal anti-GFAP antibody (Dako, Glostrup, Denmark). The sections were then incubated for 2 h with horseradish peroxidase-linked donkey anti-rabbit IgG (GE Healthcare UK). The peroxidase was visualized using 0.05% diaminobenzidine hydrochloride and 0.005% hydrogen peroxide.

For fluorescence immunohistochemistry, the brains were cryoprotected in 30% sucrose before being frozen in powdered dry ice. Coronal sections (20 µm) were cut and mounted on slide glass. The sections were then heated in 10 mM citrate buffer (pH 6.0) for 15 min at 97°C, followed by treatment with 1% Triton X-100, 2× Block Ace solution, and 10% NDS for 1 h, respectively. The sections were then incubated overnight at room temperature with mouse monoclonal antibody against GFAP and Iba1 (Millipore, Billerica, MA, USA) or rabbit polyclonal antibody against 11βHSD1 (LifeSpan Biosciences, Seattle, WA, USA). After incubation, the sections were incubated for 3 h at room temperature with Alexa 488-conjugated donkey anti-mouse IgG or Alexa 594-conjugated donkey anti-rabbit IgG (Life Technologies, Grand Island, NY, USA), and mounted using an antifade reagent containing DAPI (Life Technologies). The fluorescence signals were analyzed with a deconvolution fluorescence microscope system (Keyence, Osaka, Japan).

### Statistical analysis

Values are represented as the mean ± S.E.M. Individual between-group comparisons were employed using the unpaired *t* test.

## Results

### Autoradiography of [^3^H]GA in brain tissue

**Figure 2:**
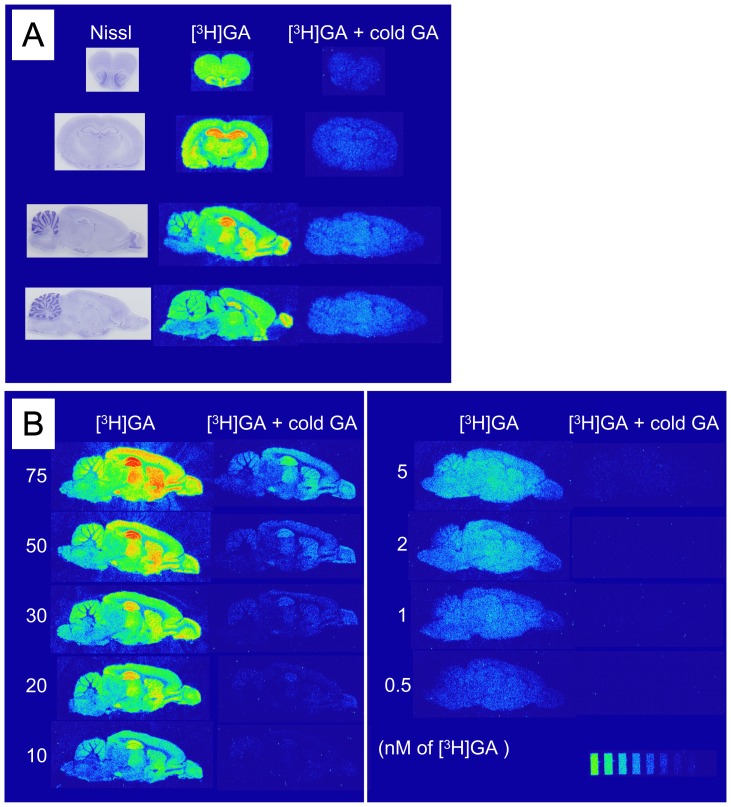
Autoradiograms of [^3^H]GA binding in brain tissue. (A) Representative autoradiograms of [^3^H]GA binding. [^3^H]GA bound several brain regions, and these bindings were clearly decreased in the presence of cold GA. (B) Dose-dependency of [^3^H]GA binding. [^3^H]GA binding was increased with rising [^3^H]GA concentration, and these bindings were clearly decreased in the presence of cold GA.

Autoradiograms of [^3^H]GA binding in rat brain tissues are shown in Figure 2. [^3^H]GA radioactivity was detected throughout the brain. These bindings were almost abolished in the presence of cold GA. Moreover, the binding increased with rising [^3^H]GA concentrations.

### Saturation curve and Scatchard plot analyses

**Figure 3:**
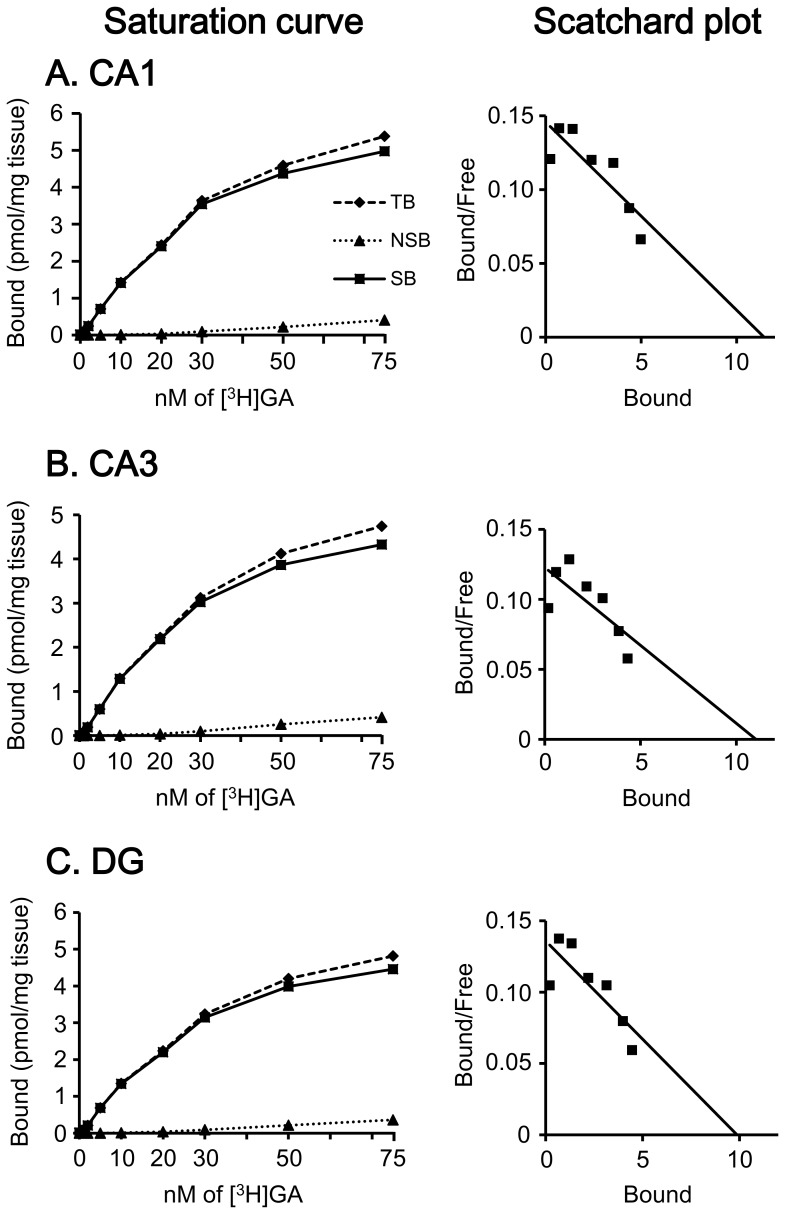
Binding characteristics of [^3^H]GA in the hippocampal CA1 (A) and CA3 (B) subfields and DG (C). Left panels are saturation curves and right panels are Scatchard plots. Each point is the mean of three independent values.

The binding characteristics of [^3^H]GA in the hippocampal formation are shown in Figure 3. In the saturation curve analysis of [^3^H]GA binding in the CA1 subfield (Figure 3A), TB was gradually increased with rising [^3^H]GA concentration, these bindings were markedly decreased by cold GA (NSB), and apparent SB was observed. The Scatchard plot analysis indicated that the regression line was linear. Very similar results were obtained in the CA3 subfield (Figure 3B) and dentate gyrus (DG; Figure 3C).

*B*_max_ and *K*_d_ values calculated from a Scatchard plot of brain regions examined are listed in Table 1. The higher *B*_max_ was observed in the hippocampal CA1 and CA3 subfields and DG. The moderate *B*_max_ was observed in the amygdala, caudate putamen (CPu), nucleus accumbens (NAcc), substantia nigra (SN), ventral posteromedial thalamic nucleus (VPM), olfactory bulb (OB), medial prefrontal cortex (mPFC), prelimbic cortex (PrL), agranular insular cortex (AI), and orbitofrontal cortex (oFC). The lower levels were seen in the dorsal raphe nucleus (DR), fifth cerebellar lobule (5Cb), and simple lobule A (SimA). In addition, many regions showed relatively larger *K*_d_ values, i.e., minimum, 64.0 nM for DG; maximum, 202.1 nM for VPM.

**Table 1:** [^3^H]GA binding in rat brain.

	*B*_max_ (pmol/mg tissue)			*K*_d_ (nM)		
mPFC	4.6	±	0.9	97.1	±	22.2
PrL	4.7	±	0.3	90.8	±	12.4
AI	5.1	±	0.9	103.6	±	22.0
oFC	6.3	±	0.7	113.2	±	21.0
CA1	11.3	±	1.5	75.3	±	14.6
CA3	11.0	±	1.9	90.8	±	26.8
DG	9.2	±	0.7	64.0	±	7.9
VPM	7.7	±	1.0	202.1	±	25.9
Amygdala	6.2	±	0.8	126.8	±	25.5
OB	4.2	±	0.2	58.3	±	3.8
CPu	6.8	±	1.3	94.8	±	18.4
NAcc	6.3	±	0.2	68.3	±	6.1
SN	7.7	±	2.3	156.9	±	56.7
DR	2.8	±	0.3	106.1	±	14.9
5Cb	3.0	±	0.4	82.7	±	21.8
SimA	3.5	±	0.6	109.0	±	29.0

Data are expressed as mean ± SEM (n = 3).

### Competition for [^3^H]GA binding by chemical compounds

**Figure 4:**
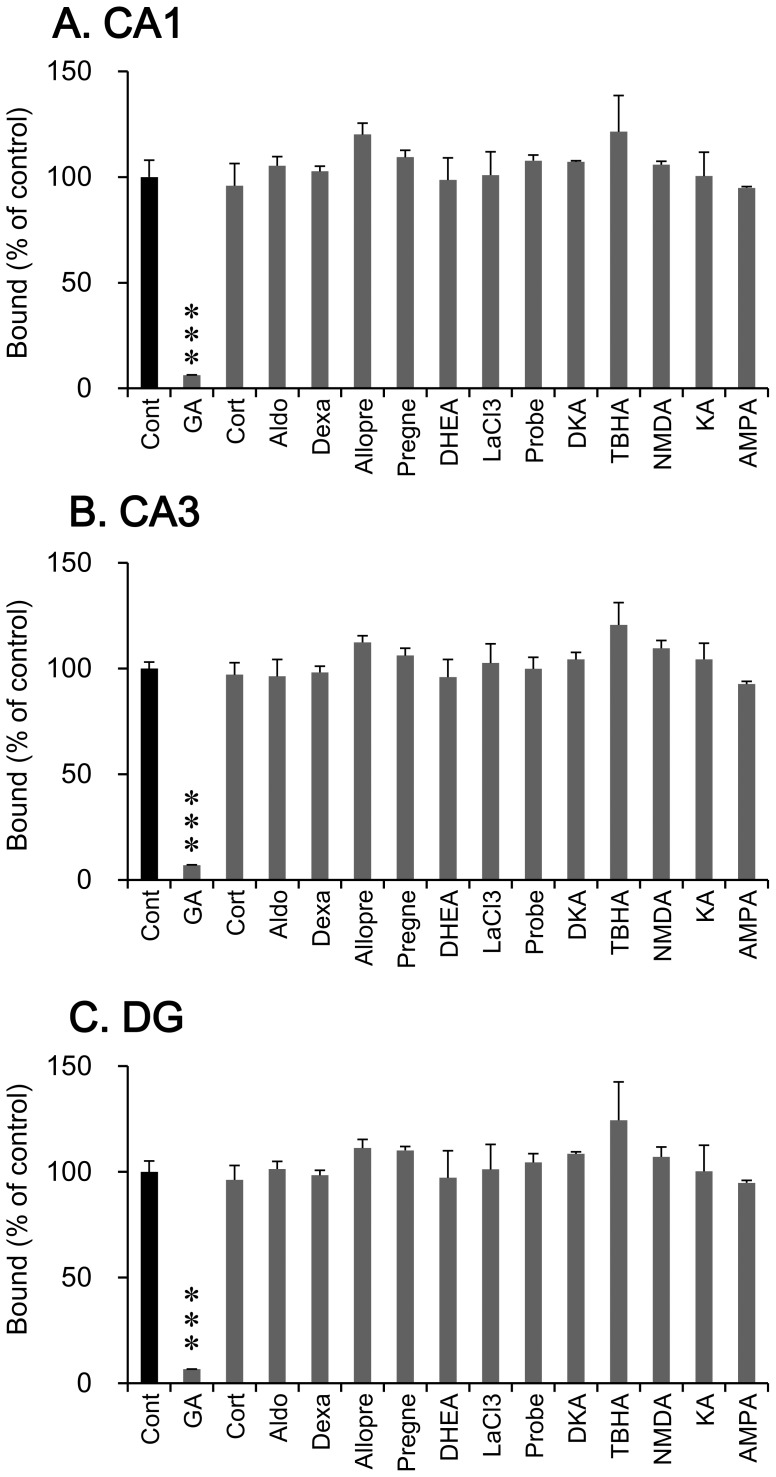
Inhibition of [^3^H]GA binding by chemical compounds in the hippocampal CA1 (A) and CA3 (B) subfields and DG (C). [^3^H]GA binding was not significantly inhibited by any chemical compound tested in each hippocampal region. Each column is the mean ± SEM (n = 3). Asterisk indicate a significant difference; ^***^ *p*<0.001 vs. control (total binding of [^3^H]GA).

In the competition analysis (Figure 4), [^3^H]GA binding in the hippocampal formation was significantly decreased by cold GA (CA1, t = 11.77, df  = 4, *p*<0.001; CA3, t = 30.27, df  = 4, *p*<0.001; DG, t = 18.05, df  = 4, *p*<0.001), but not significantly decreased by any chemical compound tested. Similar results were obtained in other brain regions such as CPu and NAcc (data not sown).

### Cells labeled by [^3^H]GA are similar to astrocytes expressing 11β-HSD1

**Figure 5:**
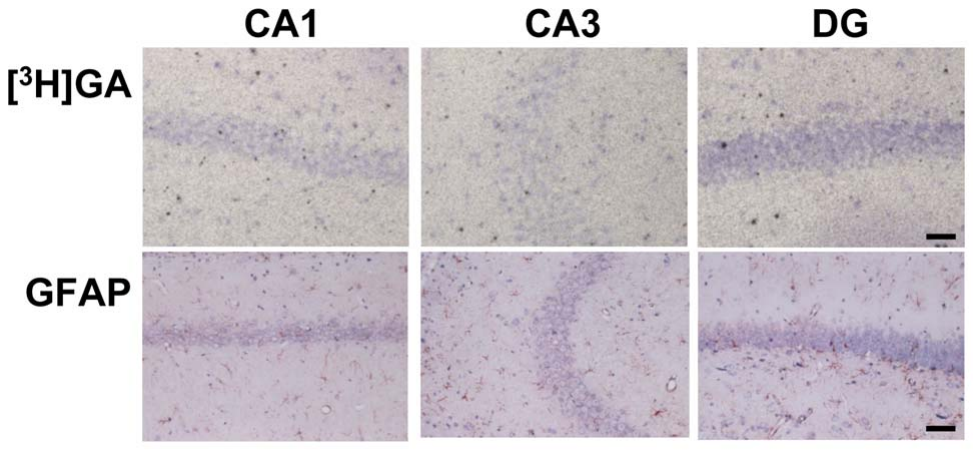
Representative microautoradiograms of [^3^H]GA binding and bright field immunohistochemical images for GFAP in the hippocampal CA1 and CA3 subfields and DG. The distributions of [^3^H]GA signals are consistent with those of astrocytes. Scale bars, 50 µm.

[^3^H]GA microautoradiograms (Figure 5) indicate that the positive signals of [^3^H]GA were detected in small cells in and around the pyramidal neuronal layer of the CA1 and CA3 subfields and the granule cell layer of the DG. GFAP immunoreactivity (Figure 5) was detected in small cells in the hippocampal formation, and their distribution was very similar to that of [^3^H]GA signals.

**Figure 6:**
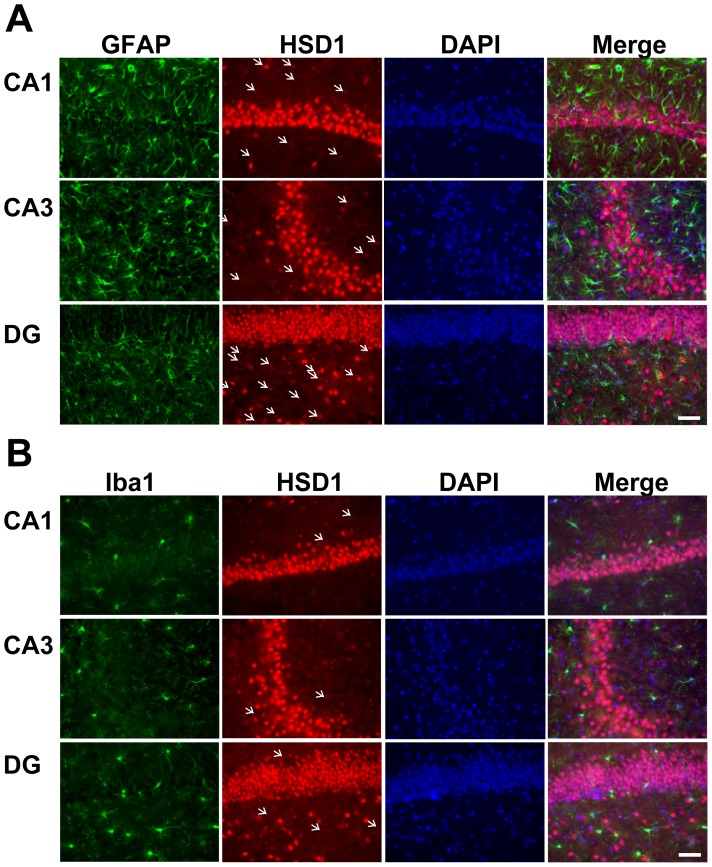
Representative immunofluorescence images for GFAP (green) (A), Iba1 (green) (B), 11β-HSD1 (red) (A and B), nucleus (DAPI, blue) (A and B), and merged in the hippocampal CA1 and CA3 subfields and DG (A and B). These images confirmed the localization of astrocytes (GFAP-positive cells), microglial cells (Iba1-positive cells), 11β-HSD1, and nuclei. Arrows indicate astrocytes (A) or microglial cells (B) having 11β-HSD1. Scale bars, 50 µm.

In fluorescence immunohistochemistry (Figure 6A), the distribution of GFAP immunoreactivity was consistent with the results of blight field immunohistochemistry. 11β-HSD1 immunoreactivity was mainly detected in the pyramidal neurons of the CA1 and CA3 subfields and was slightly, but significantly, detected in small cells around these neurons. Similarly, in the DG, the immunoreactivity was detected in the granule cells and in small cells around these cells. Moreover, merged image revealed that several 11β-HSD1 signals were colocalized with GFAP. Iba1 immunoreactivity (Figure 6B) was also detected in and around the pyramidal neuronal and granule cell layers. Merged image revealed that a few 11β-HSD1 signals were colocalized with Iba1 in each hippocampal region, but the intensity of 11β-HSD1 signals in Iba1-positive cells was weaker than that in GFAP-positive cells.

## Discussion

The main finding of the current study is that specific binding sites for GA exist in brain tissues. Moreover, the cells recognized by [^3^H]GA are thought to be astrocytes. This is the first report showing the specific binding of GA in the brain.

### Specific binding sites of GA in brain

The finding that almost all [^3^H]GA bindings in the brain were abolished by cold GA (Figure 2) indicates that specific binding sites for GA exist throughout brain tissues. This was further confirmed by the dose-dependent and saturable increase in the specific [^3^H]GA bindings (Figures 2 and 3). Subsequent Scatchard plot analysis suggests that the [^3^H]GA binding was a one-site binding manner, but *K*_d_ values were different in each region (Table 1), suggesting that [^3^H]GA binds to only molecular species, but each region has a different species. These data are substantially consistent with a previous report demonstrating that [^3^H]GA bound specifically to rat liver tissue concomitant with a *B*_max_ of 43 pmol/mg protein and *K*_d_ value of 31 nM [22].

### Possible characteristics of molecules and cells that bind GA

A previous report [6] showed that GA certainly bound to MR and slightly to GR, in cytosolic preparations of rat kidney. In brains, MR and GR are abundantly distributed in the pyramidal neurons and granule cells of the hippocampal formation [23]. It is well known that aldosterone binds to MR specifically, corticosterone binds to MR and GR, and dexamethasone binds to GR. In our study, however, these steroids, including several neurosteroids, did not compete with [^3^H]GA binding in the hippocampus (Figure 4). This inconsistency may be due to the differences in analytical methods and tissues examined: binding of GA to MR and GR was reported for a cytosolic preparation of kidney [6] while we found no binding in brain slices.

Gap junctions are channel-forming structures between the membranes of two abutting cells, and are composed of two adjacent hemichannels [24]. These molecules are expressed in not only neurons, but also astrocytes, microglia, and oligodendrocytes, in which several different types of gap junction components are expressed [20], [25]. It is well known that GA is a potent non-selective blocker of gap junctions [26], and slows neuronal oscillations and attenuates epileptic discharges and glutamate-induced neurotoxicity [16], [27] – [28]. Thus, a gap junction is a candidate target molecule for GA in the brain. Unexpectedly, in our study, [^3^H]GA binding in the hippocampus was not attenuated by gap junction blockers, i.e., La^3+^ and probenecid (Figure 4). Considering that GA can inhibit gap junction functionally, the interpretation of our data would be limited. Thus, our results suggest that GA does not bind to the sites recognized by La^3+^ and probenecid in the gap junction components, and do not rule out the possibility that GA interacts with gap junction directly or indirectly.

Interestingly, the distribution of [^3^H]GA binding sites was almost coincident with that of GLT-1, which expresses specifically in astrocytes [29], and partially with that of glutamate-aspartate transporter (GLAST) [30]. Functionally, GA increases the reduced expression of GLAST in cultured astrocytes subjected to thiamine deficiency [15]. Moreover, GA prevents glutamate-induced neurotoxicity in primary cultured neurons [16]. These findings led the possibility that GA interacts with GLAST as well as GLT-1 or several types of glutamate receptors. However, the chemicals that inhibit these transporters (DKA and TBHA) or glutamate receptor agonists (NMDA, KA, and AMPA) did not inhibit the [^3^H]GA binding in brain regions including hippocampus (Figure 4), suggesting that GA does not bind directly to GLT-1, GLAST, or glutamate receptors involving neurotoxicity.

Our microautoradiographic and immunohistochemical studies (Figure 5) revealed that [^3^H]GA-labeled cells were distributed to sites similar to astrocytes. Importantly, [^3^H]GA-labeled cells were not distributed in the pyramidal neurons and granule cells, indicating that these neurons are not the target for GA. Previously, Irie *et al* [7] demonstrated that a specific protein binding GA in rat liver is 11β-HSD1, which has a *K*_d_ value of 28 nM, indicating that 11β-HSD1 is a useful marker of cells recognized by GA in the brain. Our immunofluorescence study (Figure 6) revealed that 11β-HSD1 was dominantly expressed in the pyramidal neurons and granule cells, which was not consistent with the distribution of [^3^H]GA-labeled cells. However, 11β-HSD1 was also distributed in a large number of astrocytes and a few microglial cells, as well as other unidentified cells lacking GFAP and Iba1, in the hippocampal formation. It is possible that 11β-HSD1 is expressed in un-activated astrocytes, because GFAP sometimes does not express in un-activated astrocytes. These findings suggest that several types of cells may be included in [^3^H]GA-labeled cells, but astrocytes could be a major target for GA. In mouse brain, 11β-HSD1 mRNA was detected in and around the pyramidal neuronal layer and granule cell layer of the hippocampal formation [31], which is partially consistent with our data. Thus, we suggest that GA mainly binds to 11β-HSD1 in astrocytes in a cell-specific manner.

### Neuropharmacological relevance

The regions labeled by [^3^H]GA (i.e., the hippocampus, CPu, NAcc, amygdala, SN, and VPM) form a neural network to regulate anxiety/fear responses, as well as learning and memory, suggesting that GA is involved in regulation of emotion and cognition in its interactions with these regions. The finding that the candidate target cell for GA is astrocytes gives the importance of astrocytes in GA actions. Moreover, possible GA actions in the brain may become obvious when astrocytes are activated. For example, in humans or rodents, GFAP-positive astrocytes increase at brain sites where inflammation occurs and around senile plaques [32], [33], [34], as well as in the regions subjected to ischemia/reperfusion injury [35], and even in normally aged brain [36], [37], [38]. Our idea is supported by recent studies that astrocyte abnormalities are associated with the pathophysiology of schizophrenia [39], mood disorder [40], and Alzheimer's disease [41] and that GA acts directly upon cultured astrocytes [15]. In addition, the Glycyrrhiza-containing Kampo medicine yokukansan ameliorates BPSD in dementia patients [12] and emotinal disturbances and cognitive deficits in animals [42], [43], [44].

11β-HSD1 catalyzes conversion of active glucocorticoids (11-oxo steroids) to inactive 11-keto steroids in the presence of NADP^+^ by its dehydrogenase activity [45]. In the presence of NADPH, this enzyme shows 11β-reductase activity that is the reverse of dehydrogenase activity. NADPH oxidase activation that increases the NADP^+^/NADPH ratio is commonly involved in inflammatory reactions, and mediates oxidative stress [46]. In neuroinflammation, astrocytes are generally activated, and excessive glucocorticoids are required to ameliorate the inflammation, but 11β-HSD1 works as dehydrogenase that inactivates glucocorticoids. Under these conditions, GA can inhibit the dehydrogenase activity of 11β-HSD1 in astrocytes, leading to relatively enhanced glucocorticoid actions. Thus, we assume that GA may be more effective in emergency environments than in a steady normal state. This unique anti-inflammatory action would be commonly expressed in Glycyrrhiza-containing drugs including yokukansan.

## Conclusion

We showed evidence that specific binding sites for GA exist in the rodent brain, in which the higher density region is the hippocampus, the moderate is the CPu, NAcc, amygdala, thalamus, cerebral cortex, and OB, and the lower is the cerebellum and brain stem. In addition, a major target cell of GA is suggested to be the astrocyte having 11β-HSD1. These findings are important to understand the pharmacology of GA in the brain after oral administration of Glycyrrhiza as well as the Glycyrrhiza-containing Kampo medicine yokukansan.
